# Ethical Analysis of the Application of Assisted Reproduction Technologies in Biodiversity Conservation and the Case of White Rhinoceros (*Ceratotherium simum*) Ovum Pick-Up Procedures

**DOI:** 10.3389/fvets.2022.831675

**Published:** 2022-05-03

**Authors:** Pierfrancesco Biasetti, Thomas B. Hildebrandt, Frank Göritz, Robert Hermes, Susanne Holtze, Cesare Galli, Giovanna Lazzari, Silvia Colleoni, Ilaria Pollastri, Maria Michela Spiriti, Jan Stejskal, Steven Seet, Jan Zwilling, Stephen Ngulu, Samuel Mutisya, Linus Kariuki, Isaac Lokolool, Patrick Omondo, David Ndeereh, Barbara de Mori

**Affiliations:** ^1^Department of Reproduction Management, Leibniz Institute for Zoo and Wildlife Research, Berlin, Germany; ^2^Ethics Laboratory for Veterinary Medicine, Conservation, and Animal Welfare, University of Padua, Padua, Italy; ^3^Department of Veterinary Medicine, Freie Universität, Berlin, Germany; ^4^Avantea, Laboratory of Reproductive Technologies, Cremona, Italy; ^5^Department of Comparative Biomedicine and Food Science, University of Padua, Legnaro, Italy; ^6^ZOO Dvůr Králové, Dvůr Králové nad Labem, Czechia; ^7^Ol Pejeta Wildlife Conservancy, Nanyuki, Kenya; ^8^Kenya Wildlife Service, Nairobi, Kenya

**Keywords:** assisted reproduction technologies (ART), ethical analysis, white rhinoceros (*Ceratotherium simum*), biodiversity conservation, Ethical Matrix, ovum pick-up, northern white rhinoceros, conservation breeding programs

## Abstract

Originally applied on domestic and lab animals, assisted reproduction technologies (ARTs) have also found application in conservation breeding programs, where they can make the genetic management of populations more efficient, and increase the number of individuals per generation. However, their application in wildlife conservation opens up new ethical scenarios that have not yet been fully explored. This study presents a frame for the ethical analysis of the application of ART procedures in conservation based on the Ethical Matrix (EM), and discusses a specific case study—ovum pick-up (OPU) procedures performed in the current conservation efforts for the northern white rhinoceros (*Ceratotherium simum cottoni*)—providing a template for the assessment of ART procedures in projects involving other endangered species.

## Introduction

Assisted reproduction technologies (ARTs) offer increasingly important opportunities for biodiversity conservation (1–3). Originally applied mainly on domestic and lab animals, ARTs have found usage also in conservation breeding programs, where they can enhance the genetic management of populations, and increase the number of offspring per generation. More elaborate and costly techniques, *advanced* assisted reproduction technologies (aARTs) not commonly employed on livestock and laboratory animals, may even spark hope for the survival of taxa that are functionally extinct or at the verge of extinction (4, 5).

However, the application of ARTs in biodiversity conservation opens up new ethical scenarios that have not yet been fully explored. Like any other technology capable of redefining the boundaries of extinction (6), ARTs question the very idea of conservation we want to pursue and the values it needs to convey. Moreover, ART applications may have ethically relevant consequences—on conservation projects, on the people involved or otherwise affected, and on the animals on which they are performed—that should be carefully discussed.

The ethical assessment of the involved procedures is an integral and crucial part of the ethical assessment of conservation projects (7). Here, we propose a frame for the ethical analysis of ART procedures in conservation using the Ethical Matrix (EM), and we discuss a case study based on ovum pick-ups (OPUs) performed for the current conservation efforts of the northern white rhinoceros (NWR, *Ceratotherium simum cottoni*, Lydekker, 1908).

The NWR is a subspecies of the white rhino (*Ceratotherium simum*, Burchell, 1817) of which only two females remain (8), and whose fate is irremediably tied to the recovery and manipulation of the existing biomaterials. It should be noted, however, that the entire *Rhinocerotidae* family, consisting of five extant species—white rhinoceros, black rhinoceros (*Diceros bicornis*, Linnaeus, 1758), Sumatran rhinoceros (*Dicerorhinus sumatrensis*, Fischer, 1814), Javan rhinoceros (*Rhinoceros sondaicus*, Desmarest, 1822), and the greater one-horned rhinoceros (*Rhinoceros unicornis*, Linnaeus, 1758)—is currently under severe threat due to habitat loss and persistent poaching (9). In particular, black, Sumatran, and Javan rhinoceros are critically endangered—with the latter two species reduced to small (>80 individuals and 46–66 individuals, respectively) dwindling populations (10, 11). Moreover, even the less endangered taxon—the southern white rhinoceros (SWR, *Ceratotherium simum simum*, Burchell, 1817)—while “only” near threatened in the wild (12), does not have self-sustainable captive populations (13). It is likely that, among other strategies, future conservation efforts of rhinoceros will resort to ARTs (5). While new technologies like stem cell-associated techniques and *in vitro* follicular growth (5) may eventually ensure a stable supply of gametes without the need for *in vivo* collection, in the near future, procedures like OPU and semen collection will presumably remain the only viable methods to obtain the necessary biomaterial for *in vitro* embryo production. It is necessary, then, to analyze the ethical issues associated with these interventions.

The purpose of this study is, therefore, three-fold: (i) to provide a methodology for the ethical analysis of ART procedures in conservation projects; (ii) to use this methodology to assess the OPU procedures performed in the case study; (iii) to provide a template for the assessment of OPU procedures in other projects involving white rhinoceros or other members of the rhinocerotidae family.

## Materials and Methods

### Assessing ARTs in Conservation Projects

In human medicine, ARTs are usually defined as those procedures or treatments in which both the male and female gametes or embryos are manipulated *in vitro* to achieve pregnancy (14). In contrast, in veterinary medicine, the catalog of ART is normally broader, including, for instance, artificial insemination (15–21), cloning *via* somatic cell nuclear transfer (3, 22–25), and gamete production from induced pluripotent stem cells (3, 5). Following this broader use, the term ART will hereinafter be applied to any procedure involving, in one or more of its stages, the manipulation of reproductive cycles, gametes, or embryos with the final aim of producing a new individual.

With biodiversity conservation, we mean, instead, those scientifically grounded activities aimed at managing natural environments, ecosystems, wildlife, flora, biotic process, and, more generally, the whole biosphere with the end of maintaining and, eventually, restoring, the natural diversity of life on our planet and its evolution processes at all biological levels—from the ecosystem to genes. Biodiversity conservation is an ethically significant activity since it preserves the source of different kinds of values, both instrumental and non-instrumental.

Applications of ARTs in livestock, laboratory animals, and wildlife usually differ in their goals. In livestock and laboratory animals, ARTs are primarily used to maximize the offspring from genetically desired individuals. Producing large numbers of individuals with certain recurring genetic characteristics is instead generally neither useful nor desirable in the context of wildlife conservation. Rather, the goal of what could be termed “conservation ARTs” is to assist in the establishment of self-sustaining populations for reintroduction or as a genetic reserve. ARTs can contribute to this goal in two complementary ways. They can help increase the number of individuals in each generation, by expanding the opportunities and chances for achieving pregnancy. Moreover, they can improve the genetic management, by facilitating the breeding between spatially separate animals without the need for translocation, and by reintroducing into the gene pool those individuals who, for various reasons, are incapable of mating or breeding—including dead individuals whose suitable biomaterials have been cryopreserved.

Ethical analysis is crucial when conservation ARTs are involved. ART procedures in wildlife, for instance, are usually less established and—in some cases—more demanding for the subjected animals than those performed on the domestic animals. Moreover, given the different goals, some of the techniques used in conservation are more complex, as well as more challenging in terms of equipment and veterinary expertise required, than those normally employed for livestock. Finally, by redrawing the boundaries of the concept of reproduction—and, in some cases, of extinction—conservation ARTs can have a social and scientific impact that must be scrupulously considered.

### The Frame for the Ethical Analysis of Conservation ARTs

Ethical analysis permits us to determine whether a procedure is acceptable according to certain standards of value and to identify the critical issues that need to be addressed before its implementation. This should not be confused with the assessment of the project, or with the assessment of the specific implementations of the procedure. In the first case, the focus is much broader. In the second case, there is the need to include the various contextual variables in the evaluation. In both cases, however, the ethical analysis of the procedures provides a fundamental support: as an essential part of project assessment, and as a backbone for the assessment of implementations.

Carrying out a comprehensive ethical analysis of a specific conservation ART procedure means identifying and gathering numerous relevant factors beyond the technical and scientific details of its execution. The procedure has to be considered in the context of the project it is part of, and in the broader perspective of biodiversity conservation. Moreover, as conservation activities take place at the crossroad between different value dimensions (26), the procedure has to be evaluated in its wider effects on animals and people, that is, beyond its mere conservation value.

The factors to be considered for conducting a thoughtful ethical analysis of conservation ARTs can be grouped into five categories. One category revolves around the immediate context of the procedure, that is, around the project it belongs to, its goals, the probability of achieving them, and the values they convey. Some questions to be raised in this regard are as follows: What are the goals of the project? Have success criteria been clearly defined? How reasonable are the chances of success of the project according to these criteria? What is the conservation value of the project? What other values are brought forward by the project? In case of failure, would the project still lead to some kind of valuable advancement (ecological, scientific, social, etc.)? An exhaustive answer to the above questions would require a detailed analysis of the overall project, and is therefore not feasible when assessing a procedure. However, it is still necessary to have a sufficiently defined picture of the ultimate reasons why the procedure is undertaken, as this provides the context for assessing eventual critical aspects.

Moreover, it is necessary to focus on the role of the procedure in the project and its effectiveness in reaching the assigned goals. What purpose does the procedure serve in the project? Is the success of the procedure a key part of the project? Can there be alternatives in case of failure? Is it the most effective way to perform the task assigned? Have the alternatives been considered? How has the procedure been chosen? Besides the reasons for efficiency, the effectiveness of a procedure is a central issue where ethically relevant risks or costs are present. Moreover, the reasons that led to the inclusion of the procedure into the project should also be made explicit and examined to detect eventual biases.

The procedure must also be analyzed beyond its immediate contribution to the project. This means investigating its possible value beyond its effectiveness in carrying out the specific goal of the project. For instance, what is the scientific value of performing the procedure? Can it lead to scientific and technological improvements? Does it establish or refine protocols that could be employed in other biodiversity conservation projects? Can carrying out the procedure have a positive impact on the welfare of the animals involved? Can it have a positive social effect of some kind, for example, by promoting knowledge transfer or capacity building? While procedures do not happen in a vacuum, meaning that their implementation always happens in a project, the project itself may not exhaust their usefulness. Answering the above questions permits us to extend our understanding of the possible merits of the procedure beyond its instrumental value for the project.

Special attention should also be paid to the risks and costs associated with the procedure itself. What are the known risks of performing the procedure? Who is responsible? Can the procedure harm the welfare of the animals involved? Does it put at risk their lives? Are there risks for people? What could be the repercussions in case of failure? Are there any negative side effects to consider in case of success? As veterinary interventions, conservation ARTs invariably entail some risks during their performance as well as before and after (translocation, handling, restraining, recovery, etc.). These risks should be investigated and their distribution among the different involved stakeholders should be made clear, since this, alongside the distribution of benefits, is important to evaluate the acceptability of the procedure.

The last category of ethically relevant factors focuses on how the procedure fits into the values and worldview of public opinion and conservationists. Does the procedure raise public concerns? Are there any groups that particularly oppose it? Why? How does the procedure match or challenge the various existing perspectives on biodiversity conservation? Public opinion can be skeptical of the project and the employed procedures. Sometimes this is just due to lack of involvement or inadequate information. However, in other cases, the reasons can be more substantial: the unfair distribution of the costs and benefits of the project among the people and communities involved; there is distrust for the individuals or the institutions carrying out the project; the goals and the methods of the project conflict with the shared values, etc. Similarly, uses of conservation ARTs may challenge the tenets of some conservation philosophies. A careful analysis of the factors in this category allows for the anticipation of potential conflicts so that it should be possible to take countermeasures.

### Gathering Factors Through the EM

Table 1 summarizes the necessary factors to be considered for analyzing the applications of conservation ARTs. Some factors (i.e., the goals of the project, feasibility, and the effectiveness of the procedure) can be retrieved from the description of the project itself. Other factors must instead be identified by analyzing the procedure from an ethical standpoint. To achieve this goal, a specific ethical tool—the EM—can be applied.

**Table 1:** Relevant factors for the ethical analysis of conservation ARTs.

**Group**	**Factors to be investigated**	**Examples of associated questions**
1. Context of the procedure	• Goals of the project • Values conveyed by the project's goals • Feasibility of the project	• What are the goals of the project? • Have success criteria been clearly defined? • How reasonable are the chances of success of the project according to these criteria? • What is the conservation value of the project? • What other values are brought forward by the project? • In case of failure, would the project still lead to some kind of advancement (ecological, scientific, social, etc.)?
2. Role of the procedure in the project	• Value of the procedure for the project • Effectiveness	• What purpose does the procedure serve in the project? • Is the success of the procedure a key part of the project? • Can there be alternatives in case of failure of the procedure? • Is it the most effective way to perform the task assigned? • Have alternatives been considered? • How has the procedure been chosen?
3. Value of the procedure beyond the project	• Scientific value • Conservation value • Animal welfare value • Social value	• What is the scientific value of performing the procedure? • Can it lead to scientific and technological improvements? • Does it establish or refine protocols that could be employed in other biodiversity conservation projects? • Can carrying out the procedure have a positive impact on the welfare of the animals involved? • Can it have a positive social effect of some kind, for example by promoting knowledge transfer or capacity building?
4. Risks and costs of the procedure	• Known risks of the procedure, and their distribution • Costs of failure of the procedure • Negative side-effects of the procedure in case of success	• What are the known risks of performing the procedure? • On who do they fall? • Can the procedure harm the welfare of the animals involved? • Does it put at risk their lives? • Are there risks for people? • What could be the repercussion in case of failure? • Are there any negative side-effects to consider in case of success?
5. Views on the procedure	• Public opinion's views on the procedure • Conservationists' views on the procedure	• Does the procedure raise public concerns? • Are there any groups that particularly oppose it? • Why? • How does the procedure match or challenge the various existing perspectives on biodiversity conservation?

The EM permits us to unpack and analyze the ethically relevant aspects involved in a complex scenario, reorganizing them into a transparent and comprehensible picture of value demands. Originally developed by Mepham (27) for the ethical assessment of technologies and policies in agriculture and food processing, the EM has since been applied in many other fields—including veterinary medicine (28, 29), forestry (30), aquaculture (31, 32), assessment of human–animal interactions (33, 34), management of contaminated agricultural ecosystems and radioactive waste (35, 36), and conservation (37).

The EM embraces a pluralistic ethical approach. Cells from the first column of the EM list stakeholders. Cells from the first row list three general ethical principles, influential, recognized, and shared tenets of ethical reasoning and common morality such as wellbeing, autonomy, and fairness (38, 39). Intersecting cells list the value demands for the stakeholders derived from the general ethical principles.

The EM specifically tailored for conservation (40) includes three categories of potential stakeholders: ecological entities, individual animals, and people. Table 2 recaps the general value demands generated by applying the ethical principles on these categories of stakeholders.

**Table 2:** General EM.

	**Wellbeing**	**Autonomy**	**Fairness**
Ecological entities	Conservation	Freedom from human intervention	Equal treatment in relation to conservation
Animals	Health and functioning Absence of negative affective states and allowance of positive ones	Living natural lives and expressing species-specific behaviors	Equal treatment in relation to welfare
People	Psychological and physiological welfare Sustainable social, economical, and cultural welfare	Freedom of choice Capacity to exercise the various fundamental aspects of one's own *persona* Self-determination	Equal and fair treatment

The methodology of the EM is to apply the general template on a specific case, first by identifying the stakeholders involved, and then by applying the general ethical principles in order to derive the value demands.

### The Case Study: OPU on White Rhinoceros

As a case study, we analyzed the OPU procedures performed in the recent conservation efforts to save the NWR. The case appears interesting due to several reasons. It is rather complex, with many ethically relevant issues packed together; it involves many stakeholders and multiple value dimensions, with a variety of potential value conflicts; the ART techniques employed in the project have the potential to redefine the boundaries of wildlife reproduction and extinction.

The most peculiar aspect of the case is that the NWR has been declared “functionally extinct” (8). From ~2,230 individuals in 1960 (41), the wild population of NWR has been reduced, mainly by poaching, to a few individuals by the 1980s, and presumably erased sometime after 2007 (8). During the same years, the small population kept in zoos proved to be not self-sustainable. White rhinoceros have a low reproductive rate in captivity (42). Despite various breeding attempts, only four NWR offspring were ever known to be born in this way (at the Dvůr Králové Zoo). Since the death of the last male Sudan in 2018, two females, both living at Ol Pejeta Conservancy in Kenya, have become the lasts of their kind. They are Najin, aged 32, and her offspring Fatu, aged 21.

The current conservation efforts for the NWR by the BioRescue project—an international consortium coordinated by the Leibniz Institute for Zoo and Wildlife Research in Berlin—combine aARTs and stem cell-associated techniques (43). Frozen semen from five NWR males is available, and the stored tissue could be used in the future to produce gametes by using novel technologies. Due to severe reproductive pathologies, both the remaining females cannot carry to term a pregnancy. In the case of the older female, this is due to tendon problems in the hind legs; in the case of the younger, this is due to the uterine pathology of an unknown origin. The only current way to “de-doom” the taxon is to collect their oocytes to create embryos using intracytoplasmic sperm injection (ICSI) to be transferred into SWR recipient cows.

The first point to be made here is that, despite the possible similarities, this conservation effort must not be confused with an attempt at de-extinction. De-extinction can be defined as the process of bringing back an extinct taxon (6), and it can be divided into two categories: the de-extinction of recently extinct taxa, and the de-extinction of species that had gone extinct hundreds or thousands of years ago, and whose significant ecological relationships have now disappeared [“deep de-extinction”; (44)]. Both the categories raise specific conceptual and ethical challenges (45). While de-dooming a functionally extinct taxon like the NWR may resemble a case of de-extinction in the first, non-deep, sense—in both scenarios the original ecological context still exists—the two differ in a decisive aspect, that is, generational continuity. Generational continuity cannot be recreated through de-extinction, and this may constitute in some taxa both an ecological and ethical issue: ecological, as some behaviors and functions can only be acquired through interaction between adults and juveniles; and ethical, as animal welfare may be harmed by the absence of these behaviors and functions. For these reasons, it makes sense to keep conceptually distinct the actions of de-dooming a functionally extinct taxon and de-extinguishing a vanished taxon. In the case of NWR, since only two females remain, one of which had no offspring—the generational continuity is at least partially impoverished. Nevertheless, it has not disappeared altogether, and SWR individuals can be used as proxies for NWR adults to transmit those behaviors that are known to be similar between the two subspecies, such as reproductive behaviors (46).

*In vivo* oocyte collection in rhinoceros is a relatively new intervention. The full procedure as it is performed currently in white rhinoceros involves ovarian super stimulation, full anesthesia, and transrectal ultrasound-guided oocyte recovery (17, 43, 47). In the addressed context, the procedure has been conducted regularly (albeit with at least 3 months of lapse) in the two remaining NWR females (48). Table 3 recaps the results of the seven procedures that have been executed so far in NWR. Overall, the procedure has been rather successful in Fatu, with 95 oocytes collected in seven OPUs between 2019 and 2021, which have been used to produce a total of 13 embryos. The procedure has been less successful with Najin presumably due to her age and health, and the partners in the project have decided to discontinue performing OPU on her in 2021. Although this choice further reduces the gene pool available for embryo creation, it was preferred over other options after carefully considering the ethical and scientific elements involved (49).

**Table 3:** Results of OPU and ICSI on NWR.

	**Najin**		**Fatu**	
	**Oocytes**	**Embryos**	**Oocytes**	**Embryos**
1. (08/22/2019)	5	0	5	2
2. (12/17/2019)	3	0	6	1
3. (08/18/2020)	2	0	9	0
4. (12/13/2020)	0	–	14	2
5. (03/28/2021)	–	–	21	4
6. (07/06/2021)	–	–	17	3
7. (10/25/2021)	–	–	23	1
**Total:**	10	0	95	13

At the same time, SWR oocytes are also collected from females across European zoos, in order to establish the technology also for this taxon and to synergistically support the research related to the project.

## Results

### Building Up the EM

Following the proposed methodology, an EM for the procedure has been developed (Table 4) using the template provided in Table 2. The stakeholders included in the EM are *biodiversity*, the individual females subjected to the procedure, and *all people involved in the project*.

**Table 4:** EM for OPU in NWR conservation efforts.

	**Wellbeing**	**Autonomy**	**Fairness**
Biodiversity	*Conservation* • NWR has a historical-naturalistic value. • NWR has an ecological value. • Cryobanking is a good conservation strategy *per se* (collect now or regret later). • Refining through application the OPU procedure may open new ways for the conservation of other taxa. • Incidents or complications during the procedure could damage the image of the project and of conservation ARTs in general.	*Freedom from human intervention* • Conservation ART may be deemed a technofix. • Conservation ART may lead to moral hazard. • Conservation ART may be deemed hubristic.	*Equal treatment in relation to conservation* • Charismatic animals like rhinoceros receive a disproportionate amount of attention. • However, conservation of the NWR could benefit the conservation of other less charismatic species. • The opportunity costs of the project do not fall on more traditional conservation efforts, including conservation of other rhino taxa.
Rhino females subjected to the procedure	*Health and functioning. Absence of negative affective states and allowance of positive ones* • Some aspects of the procedure may harm the animals according to these dimensions of welfare. More specifically: ovarian superstimulation, anesthesia, transrectal puncture all bear a possible risk of side-effects.	*Living natural lives and species-specific behaviors* • The procedure increases the possibility for some of the animals involved to express social behaviors currently not accessible.	*Equal treatment in relation to welfare* • The animals involved are treated like a means for the conservation of their taxon. • However, they receive extra veterinary screening and care.
People	*Psychological and physiological welfare* • Affective value for people caring for the animals. *Sustainable social, economical, and cultural welfare* • Economic value of the animals. • Ecotourism.	*Capacity to exercise the various fundamental aspects of one's own* persona • The procedure is an opportunity for professional growth, knowledge transfer, and capacity building. • NWR may have eudaimonistic (aesthetic, scientific, and reverential) value for people. • NWR may have transformative value for people. • NWR may have existential value for people.	*Equal and fair treatment* • Costs and benefits of the procedure should be distributed equally, and compensation given whenever this is not possible.

The level of resolution of the EM could be increased by adding more stakeholders or breaking down the existing ones into more specific items. It could be possible, for instance, to break down biodiversity into the different rhino species and the ecosystems involved or to add to the list the NWR calves born as a result of the project, the conservationist community, etc. Such a high-resolution EM would be especially useful to analyze the whole project in detail. However, since the goal is to assess a specific procedure, increasing the resolution of the EM is neither necessary nor desirable.

#### Biodiversity

The three basic value demands for biodiversity are (refer to Table 2): (i) conservation (under wellbeing); (ii) freedom from human intervention (under autonomy); (iii) and equal treatment in relation to conservation, without bias grounded on human preferences (under fairness).

From the standpoint of conservation, at least three values can be attached to the goals of the project, that is, bringing the NWR population back to a viable level—attaining demographic security and stability (50)—and subsequently reintroducing the taxon into the wild. The first two values are the historical and the naturalistic values of the subspecies—being a unique and irreplaceable product of the evolutionary process which would be lost for purely anthropogenic reasons. The third value is the ecological value of this taxon. Mega-herbivores are important ecosystem engineers whose contribution to shaping their environment cannot be replicated by smaller herbivores (51). White rhinoceros make no exception to this rule, and their presence can make a difference in preserving the African savannah ecosystem (52, 53). Reintroducing the NWR would then be a way to restore and maintain the ecological relationships that are now lost.

The OPU procedure has also an additional conservation value which is independent from the succes or the failure of the project. Due to the mounting extinction crisis (54), cryobanking biomaterial from endangered taxa has become an important conservation goal (19, 55), following the imperative to collect now, or regret later (5). Moreover, by carrying out the procedure, it is possible to collect technical and scientific data for developing OPU protocols in other rhino taxa, or even in other large mammals, expanding in this way the opportunities for their conservation. However, accidents during the procedure could damage the image of the project.

From the standpoint of freedom from human intervention, this procedure, like other conservation ARTs, could be considered a negative example of “technofix” that is, the use of a technology to reverse the outcomes of morally problematic activities (in this case, poaching and habitat loss) leaving intact the causes (56). Similarly, the methodology of the project could be accused of making wildlife decline overly mundane, by providing, at least in theory, an “easy” way to revert the phenomenon. This could create a moral hazard, which, in turn, could help further accelerate the extinction crisis. Finally, applications of conservation ARTs to de-doom the functionally extinct taxa may be accused to be an aggressive form of conservation, through which we attempt to forcefully impose our scheme and solutions on reality, following a hubristic attitude which has already been shown to be a part of the problem and not of the solution.

Considering equal treatment in relation to conservation, the question may be raised as to why concentrate so much effort and resources on one rhino subspecies when there are so many other endangered taxa. Rhinoceros are among the most charismatic animals (57), and this may be an explanation, albeit one that clearly expounds a bias. However, there are good reasons for not considering the choice of the NWR as unfair. Rhinoceros can serve as umbrella and flagship species (58), meaning that the reintroduction of the NWR could foster the conservation of other less charismatic species (59). Furthermore, as previously mentioned, the refinement of conservation ART protocols could open new opportunities for the conservation of other rhino taxa or even other large mammals.

In fact, one of the advantages of this project is that its opportunity costs do not fall on other more traditional conservation endeavors, including other rhino conservation efforts. This is because it draws on funds allocated for biotechnology, and does not make use of the money collected for conservation of other rhino taxa.

#### Females Subjected to the Procedure

Table 2 lists three basic value demands for the females subjected to the procedure: (i) health and functioning and absence of negative affective states and allowance of positive ones (under wellbeing); (ii) living natural lives and species-specific behaviors (under autonomy); (iii) equal treatment in relation to welfare (under fairness). This captures the multidimensional nature of animal welfare (60) and should help in gathering useful elements for the assessment relative to the risks and costs of the procedure and its value beyond the goals of the project.

Regarding the first value demand, OPU on rhinoceros is a relatively new intervention, and, as such, there is no specific and systematic investigation of its effects, immediate or prolonged, on any of the previously defined criteria of animal welfare. An overall evaluation can nevertheless be attempted, starting with some considerations to be extrapolated from similar (yet not analogous) interventions performed on other species. OPUs have been performed regularly on domestic animals in the recent decades. *In vivo* oocyte collection was first performed on cattle *via* laparoscopy (61), and, a few years later, transvaginal ultrasound-guided follicle aspiration was introduced (62, 63). Today, laparoscopic OPU is still used in small ruminants, such as sheep and goats (64), while transvaginal ultrasound-guided OPU has become the standard for cattle, buffalo, and horses (65, 66). Applications of these methods to exotic species were first performed in the mid-nineties (67), starting with zebras (68), and llamas (69).

Transvaginal ultrasound-guided OPU procedures are regularly repeated in the same cattle and buffalo cows twice per week (66, 70–72), as this is the frequency that assures the best yield of the oocytes (65). Horses can be subjected to OPU procedures on a biweekly schedule (73). The effects of the procedure and of its steady repetition in cattle, buffalo, and horses concerning the reproductive and productive capacities of the treated animals are well-documented (65, 70, 74–76).

In this regard, there is a general consensus that OPU procedures, even when reiterated regularly and for prolonged periods of time, do not have particularly adverse side effects. Studies with a stronger focus on criteria relative to the minimization of unpleasant affective states, partly caution this optimism, highlighting some invasive aspects of the OPU procedure. While repeated transvaginal punctures seem not to provoke the signs of short- and long-term stress neither in cattle (77), nor in buffalo cows (78), other possible sources of welfare impairments are nevertheless present, namely the possibility of minor ovarian alteration, and, most importantly, the negative physiological and behavioral responses to the epidural anesthesia administered during the procedure (79, 80). Studies on the reaction of horses to transvaginal ultrasound-guided OPU in terms of pain and discomfort are few and less systematic (24), but possible negative side-effects of the procedure have been reported (81–83).

In general, the OPU procedure on rhinoceros is related to those practiced on horses and cattle (43, 47). Horses, in particular, being members of the order *Perissodactyla* like rhinoceros, are considered good models due to their taxonomic relatedness. However, two crucial differences between the specific procedures complicate any possible linear comparison: the transrectal instead of transvaginal approach, and the full anesthesia.

The length of the reproductive tract, and the impossibility of palpating the ovaries through the rectum, make the transvaginal approach unfeasible in rhinoceros (except for the Sumatran rhinoceros). Since the classic laparoscopic approach is equally unfeasible (47), OPU in rhinoceros is performed transrectally (84). This raises issues of limited sterility of the procedure and of the possibilities of infection. Indeed, even if restricted to a single penetration of the rectal wall, OPU in rhinoceros still poses a minimal risk of bacterial contamination of the puncture needle even after a prior thorough cleaning and disinfection of the rectum (47).

Moreover, safe immobilization and full anesthesia are required to perform the OPU procedure in rhinoceros. Full anesthesia prevents unexpected movement, limiting the risk of injuries both to the animal and to the people carrying out the operation, yet it poses its relevant risks of complications. Standard anesthesia protocols in rhinoceros are etorphine hydrochloride-based (85). Some of these protocols have been reported to be suitable for weekly (86) and bimonthly (87) anesthetization of the same animals—a black rhinoceros and a greater one-horned rhinoceros, respectively. Nevertheless, anesthesia in general, and the use of etorphine-based protocols in particular, have been associated with many potential and possibly fatal complications, including aspiration, respiratory depression, hypoxemia, hypertension, pulmonary shunting, and ventilation/perfusion mismatch (88–91). Moreover, etorphine can be very dangerous to people, and cases of accidental exposure, while very rare, are reported in the literature (92, 93).

Transrectal oocyte retrieval is preceded by ovarian stimulation. The ovarian stimulation protocols administered to the animals employ Histrelin, a slow-release GnRH analog. The GnRH analog is injected every other day either three or four times before the OPU procedure. Captive white rhinoceros are known to suffer from various genital tract pathologies, most likely favored by long non-reproductive periods (94). Hormonal stimulation could potentially contribute to the progression of these pathologies.

From the standpoint of the second value demand, that is, the possibility of living natural lives and expressing species-specific behaviors, the procedure, by contributing to the success of the project, could be evaluated positively, at least for the two NWR females, as it may provide them, in the medium term, with a chance for expressing some parts of their behavioral repertoire which are currently not accessible. White rhinos form cow-calf and cow-adolescent pairs, which are typical groupings in the social structure of the species, with no need for males to rearing a calf (95). This means that there is a concrete possibility that the remaining females could establish social bonds with the newborn NWR. In this regard, it is important to note that, although both Najin and Fatu were born in captivity, they were accompanied during their earlier lives by several other captive-born as well as wild-caught NWR, and had, in this way, enough opportunity to learn social behaviors from conspecifics. Although it is not possible to determine *a priori* to what degree the normal social structure of the species can be recovered from this bottleneck of two individuals, returning the population to viable numbers could allow its members to cultivate a wider range of species-specific social behaviors.

On the other hand, from the standpoint of the third value demand, equity regarding welfare would require managing similar animals in the same manner. This is violated as soon as the animal is subjected to a procedure that could cause stress, discomfort, and even, in the worst cases, harm, without any direct and substantial benefit. However, while it is undeniable that in the procedure animals are mainly treated as a means for a goal—the collection of oocytes—which is only tangentially tied to their wellbeing, it is equally true that they receive much more veterinary screening and care than what constitutes the norm for white rhinoceros in captivity. Given the particular vulnerability of captive female rhinoceros to reproductive tract pathologies, such as tumors (42, 94, 96, 97), this is not an aspect to consider lightly.

#### People Involved in the Project

Table 2 lists three basic value demands for people involved in the project: (i) psychological and physiological welfare and sustainable social, economical, and cultural welfare (under wellbeing); (ii) freedom of choice, capability to exercise the various crucial aspects of one's own *persona*, as well as self-determination (under autonomy); and (iii) equal and fair treatment (under fairness). This should help in gathering useful factors for the assessment relative to the context of the procedure, of its value beyond the project and of its risks and costs.

Considering the first value demand, it is important to note that several people—keepers, veterinarians, caregivers—have regular, if not daily, contact with the animals involved, and may have built affective bonds with them. It may be expected that these people will be especially concerned for the safety of the animals during the procedure.

A second aspect to note is that the animals involved have a certain economic value, which could be reduced in case of complications during the procedure. At the same time, communities living in the area of the eventual reintroduction of the NWR could benefit from the success of the project, as it could create new opportunities for ecotourism.

Concerning the second value demand, the possibility of performing the procedure can be both an opportunity for professional growth and, given the international nature and the cutting-edge technologies of the project, an occasion for knowledge exchange and transfer. Re-establishing a self-sufficient population of NWR and reintroducing it could also promote several kinds of values linked to our fulfillment as individuals (98, 99). Indeed, majestic animals like rhinoceros can be sources of aesthetic value, scientific value, reverential value, and transformative value—meaning with this latter, the capacity of producing powerful and even life-changing experiences. Moreover, even just knowing that the NWR has been saved from extinction can be important for many people (the so-called existential value of biodiversity), even if they cannot directly experience or benefit from this.

Concerning the third value demand, a requirement should be that costs and benefits of the procedure be distributed equally, and compensation should be given whenever this is not possible.

## Discussion

### Factors for the Assessment

Along with the results from the project description, the value demands listed in the EM can be used to gather the factors for the ethical analysis frame presented before. Table 5 shows the outcome of this process.

**Table 5:** Factors for the ethical analysis of OPU procedures in white rhinoceros.

**Category**	**Factors to be investigated**	**Description**
1. Context of the procedure	• Goals of the project • Values conveyed by the goals • Feasibility	• The ultimate goal is to create a self-sustaining population of NWR to be reintroduced into the wild. This will be the ultimate criterion of success of the project. • Such a goal conveys several form of value: - *Historical, naturalistic* and *ecological* value directly tied to saving the NWR from extinction and reintroducing it. - *Welfare* value, for giving to the two remaining NWR the chance to exercise social behaviors currently not accessible. - *Economic* value, tied to the opportunity for ecotourism. - *Transformative* value for people, as encounter with NWR could lead to life-changing experiences. - *Eudaimonistic* (*aesthetic, scientific* and *reverential*) value, as encounter with NWR could lead to significative experiences. - *Existential* value, as people could still find valuable the existence of the NWR even without directly experiencing it. • It is not possible to establish with absolute certainty that the project is inevitably destined to succeed due to the limited access to biomaterial and the cutting-edge technology it requires. • The scientific and conservation values fulfilled by the refinement of protocols could still be realized even in case of failure of the project.
2. Role of the procedure in the project	• Value of the procedure for the project • Effectiveness	• Performing the OPU procedure is needed to collect the necessary oocytes for refining the ICSI and ET protocols, defining embryo quality standards, and creating NWR embryos. For this reasons, it is a key part of the project. • While gamete production from somatic cell associated-techniques can perform a crucial complementary role to the OPU procedure, techniques are still in the process of being adapted to rhinoceros. • The OPU procedure has shown to be rather effective, with 95 oocytes retrieved so far from a single NWR female, Fatu, in 7 interventions, and 13 embryos created *via* ICSI (see Table 3).
3. Value of the procedure beyond the project	• Scientific value • Conservation value • Animal welfare value • Social value	• Beyond its instrumental value for the project, the procedure conveys several other forms of value: - *Scientific* and *conservation* values for cryopreserving biomaterial from an endangered taxon and refining new protocols that could be used for projects involving other taxa. - *Welfare* value, as extra veterinary screening and care is provided to the animals involved. - *Social* value, by fostering knowledge transfer and the development and strengthening of links between people, groups and institutions dedicated to conservation.
4. Risks and costs of the procedure	• Known risks, and their distribution • Costs of failure • Negative side-effects in case of success	• Some parts of the procedure (ovarian superstimulation, anesthesia, transrectal ovarian puncture) may lead to complications that could harm the animals involved. • Negative repercussions in case of complication could be: animal welfare impairment; economic damage to the owners; suffering to people who had established bonds with the animal; damage to the image for the project and for the entire conservation world.
5. Views on the procedure	• Public opinion's views on the procedure • Conservationists' views on the procedure	• Conservation ARTs may be accused of being a technofix, of creating a moral hazard, and of being hubristic.

#### Context of the Procedure

The ultimate goal of this conservation effort is to create a self-sustaining population of NWR to be reintroduced into its still existing natural habitat. Establishing a population with these characteristics is, therefore, the ultimate success criterion of the project. This goal conveys many kinds of values: from the historical and naturalistic to the ecological, economic, transformative, eudaimonic, and existential. Success would also provide for some of the involved females to expand their current range of accessible social behaviors.

To reach this goal, the development of technologies and protocols, not yet available (at least for rhinoceros), is required. This means that it is not possible to establish with absolute certainty that the process is inevitably destined to succeed. However, some of the values conveyed by the project would still be fulfilled even in the event of a failure. Given its use of cutting-edge technologies, for instance, the scientific value of the project will still be high even in case of failure and the accumulated knowledge could be used to establish and improve similar procedures. Moreover, there are no opportunity costs falling on traditional conservation efforts, because the project draws from funds allocated for biotechnology and does not use the money raised for the purpose of funding conservation of other rhino taxa.

However, even in the case of success, some aspects must be taken into account when providing an overall evaluation of the project. One aspect concerns the welfare of the newborn calves. Although there is no reason to think that the calves will receive less attention than other white rhinos born in captivity or residing at Ol Pejeta Conservancy, it is not possible to know, in advance, if social interaction problems may arise due to rearing issues. A second aspect to be taken into consideration concerns the possible reintroduction of the NWR into the wild. In addition to all the welfare issues that can arise during a reintroduction (100), the chances of success for the operation lie on the possibility of removing the causes that led in the first instance to the decimation of this taxon, which have to be traced primarily in poaching.

#### Role of the Procedure in the Project

The OPU procedure is a key part of the project. In the SWR females, OPUs are performed to obtain the biomaterial needed for establishing new protocols for *in vitro* embryo production *via* ICSI and embryo transfer (ET). This is fundamental both for the “de-dooming” of the NWR as well as for establishing self-sustaining captive backup populations of SWR and helping with their future conservation. In the NWR females, OPUs are performed for producing embryos to be implanted as soon as the protocols for ET are ready. Presently, no alternative exists to this method of obtaining NWR oocytes, but, in the future, gametes could be obtained also from stem cell-associated techniques (5).

#### Value Beyond the Project

Beyond its immediate use in the project, carrying out the procedure conveys scientific, conservation, welfare, and social values. The refinement of techniques and protocols, the acquisition of new data, and the recurring veterinary screening of the animals can lead to technological and scientific improvement, which, in turn, may have positive repercussions on other conservation efforts. Moreover, the collection of biomaterials from the endangered taxa for cryopreservation has a scientific and conservation value independent from the project goals, due to its insurance value—meaning with this latter expression, the value inherent in the possibility that in the future the conserved biomaterial could be used for scientific or conservation purposes in ways unknown today or not yet developed. Given the international nature of the project, carrying out the procedure fosters knowledge transfer and the development and strengthening of links between people, groups, and institutions interested in conservation.

#### Risks and Costs

The main risks of the procedure are that it may harm the animals involved. This would be a problem from the point of view of each of the three value dimensions considered: the animal welfare dimension, for obvious reasons, but also the conservation dimension, since an accident could diminish the chances of saving the taxa, and the human dimension, since many people, for various reasons, care about the wellbeing and health of the two animals.

Specifically, there are three potentially critical factors in the procedure: ovarian stimulation involves a series of injections with a GnHR agonist which may accelerate pre-existing pathologies in certain individuals; the transrectal nature of the operation, which despite all caution may lead to enhanced infection risks; general anesthesia, which, while reducing the need of mechanically restraining the animal, can give rise to complications.

In the event of a complication due to the procedure, the negative repercussions would be manifold. In addition to the harm caused to the animal involved, the possible economic damage to the owners should be considered. Other negative repercussion will be the suffering caused to people who had established relationships of some kind with the animal and the damage to the image of the project and for the entire community of conservationists.

#### Public View

The use of biotechnologies is particularly debated since, according to some, it distorts some fundamental aspects of the mission of conservation. Conservation ARTs could be accused in this sense to be a form of technofix, of creating a moral hazard, and of being a manifestation of hubris.

### Evaluating the Conflicts and Addressing the Concerns

After building up the EM and mapping the factors involved in the assessment, the main goal of the ethical analysis is to evaluate the conflicts and to address the concerns. Conservation efforts raise inevitable conflicts, as their implementation usually affects different value dimensions and has to deal with complex sets of, often, irreconcilable demands. This is the case also with the OPU procedure that we have been analyzing, especially concerning two issues: the welfare of the involved animals, and the idea of conservation it may convey.

#### Concerns for the Welfare and Lives of the Animals Involved

Actions necessary for the conservation of the NWR taxon may be detrimental, in case of an accident or complication, to the welfare of the rhinoceros involved in the project, or even pose a threat to their life. However, refusing to intervene would mean failing the duty to conserve important elements of the biodiversity of Earth.

A possible radical solution to this conflict would be to rely on an alternative biotechnology, such as the production of gametes from induced pluripotent stem cells. In this way, the same results could be obtained without the risks associated with the OPU procedure. The trouble with this solution, however, is that at the moment, this technology is not yet available for rhinoceros. Due to the age of the remaining NWR, waiting could mean losing the possibility of having both females alive when the first calf will be born, further limiting the generational transmission of skills and cultural traits. While behaviorally the NWR and SWR do not seem to differ decisively from each other, there are some unique elements in the repertoires of the two subspecies. In particular, eating habits seem to differ (46), as well as, to some extent, vocalizations (101). The role played by generational transmission in the expression of these behaviors is not clear, and it is also not clear whether they could be eventually recovered and passed to the future generations of NWR. Nonetheless, it would be unwise to miss this last opportunity, especially considering that the eldest of the two females, Najin, was able to carry out a pregnancy and rear an offspring.

The only viable solution, at present, is to reach an acceptable compromise among the different value dimensions involved. This means that no value demands can be disregarded, or on the contrary, assumed as the only important one to follow. For instance, however valuable we may consider the conservation effort for the survival of NWR, it cannot overrule the basic requirements of animal welfare. At the same time, it must be accepted that as veterinarian procedures, OPU interventions necessarily involve some level of risk concerning the life and the welfare of the animals.

Ovarian stimulation is the first potentially problematic issue of the procedure and should be avoided where there are concrete risks to promote tumor growth in the reproductive tract and induce malignancy.

A second issue is anesthesia, which can give rise to dangerous side effects or even results in the death of the animal. To cope with the matter, the OPU procedure on NWR makes use of an anesthesia protocol specifically devised (102). The main advantage of this protocol is that it is etorphine-free, preventing in this way all the possible side effects associated with this drug, which can be rather severe for the cardiovascular and respiratory systems (88–90), as well as risks of accidental exposure. The protocol is based on four different drugs (butorphanol tartrate, detomidine hydrochloride, midazolam hydrochloride, and ketamine hydrochloride), which interact synergistically with one another, enabling a reduction of their dosage and hence their possible side effects. Moreover, each of these drugs—except for ketamine hydrochloride—has an antidote, and their effects can be reversed completely.

Butorphanol-based protocols are considered a valid alternative for immobilizing white rhinos (103) and have been shown to produce less respiratory depression and hypoxia (104). Currently, this protocol has been used on more than 500 rhinoceros of different species—both in captive, wild, or semi-wild conditions—and has shown no side effects even if repeatedly used in the same individuals. Consecutive repetition of the protocol makes it possible to better tailor it to the peculiarities of the specific animal. Moreover, the unnecessary use of anesthesia—something to be avoided especially in old animals—can be minimized by proceeding with a preliminary ultrasound screening when the animal is only lightly sedated (i.e., standing sedation), and then choosing whether to continue and proceed into full recumbent anesthesia or terminate the procedure. While frequencies of the procedure similar to those in use with cattle, buffalo, and horses are ruled out, these safer anesthesia protocols allow for the repetition of multiple OPUs on the same individual within a reasonable lapse of time (4).

Finally, a third issue comes from the transrectal puncture which is required to reach the ovaries. Even if restricted to a single penetration of the rectal wall, this puncture still poses a slight risk of infection due to the potential contamination of the puncture needle (47). To mitigate this risk, the rectum of the animal is thoroughly cleaned and disinfected before the procedure, following operative standards similar to those used in human medicine prior to colon resection (47).

In order to check each application of the procedure, an ethical self-assessment through a dedicated tool, ETHAS (105), is practiced before each intervention.

Table 6 recaps all the animal welfare issues and the minimization strategies adopted.

**Table 6:** Welfare issues and minimization strategies.

**Procedure**	**Animal welfare issue**	**Minimization strategy for the con**
Ovarian stimulation	Ovarian stimulation increases the number of available follicles, helping in this way to maximize the collection of oocytes per anesthesia and reducing the number of interventions as much as possible. **Con**: Injections can be stressful for the animals. Ovarian stimulation may accelerate the progression of certain existing genital tract pathologies.	Exclusion of animals with severe genital tract pathologies from the OPU program.
Full anesthesia	Full anesthesia removes the necessity for mechanically restraining the animals during the procedure—with all the associated risks of injury. **Con**: May cause severe complications such as aspiration, respiratory depression, hypoxemia, hypertension, pulmonary shunting and ventilation/perfusion mismatch.	Specifically designed ethorphine-free protocol already tested on 500+ animals. The protocols employ four different drugs in order to lessen their individual dosages. For each drug with the exception of ketamine hydrochloridre a specific antidote is available to immediately reverse the effects. Preliminary ultrasound screening may remove possibility of unnecessary use of anesthesia. Ovarian stimulation, maximizing the number of oocyte recovery for each intervention.
Transrectal ultrasound-guided oocyte recovery	**Con**: Non sterility of the procedure, with the risk of infection.	Cleaning and disinfection of the rectum prior the procedure adopting operative standards from human medicine. Ovarian stimulation, maximizing oocyte recovery for every intervention.

#### Concerns About Conservation ARTs

Conservation ARTs push us far from a model of conservation where our main goal is to limit our interaction with the natural processes. Conservation ARTs, in fact, redefine one of the most paradigmatic of the natural processes, reproduction. In this regard, conservation ARTs may be accused to be hubristic, to be a technofix, or to create a moral hazard.

Without pretending to exhaust the complexity of these arguments, it can be nevertheless noted that they are often used to prove too much with too little. The hubris argument, for instance, is often grounded on the idea that some technologies—particularly those that, by breaking new ground, run the inevitable risk of producing unexpected consequences—may create more problems than they address, and eventually, may even lead to catastrophe. When this argument is used to urge caution, there is nothing suspicious in it, because, in applying a new technology, the risks are often real. However, if the argument is generalized to claim that every application of new technology, even when adopting the necessary measures and protocols, will produce uncontrollable negative consequences, then it is no more plausible.

Concerning the technofix argument, there could be few objections to the fact that conservation ARTs are an attempt to reverse the effects of an ongoing process, that is, human-caused extinction, through the use of technology. This remark, however, can be interpreted in two senses. In the first sense, it can be interpreted as an invitation to not lose sight of the causes that led to the current state of affairs regarding the NWR. This is important. Trying to reverse the decline of a population cannot be done without removing the original causes that led to this situation. Addressing the causes is, in this sense, a necessary condition for success. In a second sense, the previous remark can be interpreted as stating that there is something inherently wrong in working on the effects because this is not sufficient. This is misleading because something not sufficient might still be necessary. In the case of the NWR, for instance, the extinction clock cannot be brought back just by solving the issues that set it into motion, as reverting the population decline is also needed.

The moral hazard argument is based on the claim that having an easy way to revert extinction could make us even more reckless in our attitude toward biodiversity and the environment. To use an analogy, having a lifeboat at our disposal could make us more foolhardy in driving the boat. Again, if this argument is used to caution against the possible perils of new technology, it is sound. If it is used instead to convince us to abandon the technology, it is implausible. Lifeboats may make us more risk-prone, as much as car insurance is said to make drivers less prudent. However, people just do not stop using them because they might increase the risk of incidents. This is because their benefits, in case of an incident, are higher than the costs associated with the risks they may create. The same happens with conservation ARTs: their utility far surpasses the moral hazard they might pose by granting us with a certainly not easy, but nevertheless possible, way to reverse extinction.

## Conclusion

Ethical analysis provides us with a way to reflect on a procedure or on a project and it is a necessary step in making its responsible implementation possible. This study presented a frame for the ethical analysis of conservation ART procedures based on the use of the EM to collect the ethically relevant factors to identify issues and value conflicts. The advantages offered by the use of the EM are manifold. In particular, the EM makes it possible to collect and organize the elements, starting from several principles and stakeholders, allowing for a more balanced approach in evaluating complex moral scenarios where different needs, interests, and ethical concerns may conflict.

The focus of the frame presented here is on procedures, and as such, it cannot replace a structured assessment of projects. Although it includes among its requirements the analysis of the general goals and of the context of the procedure, it should not be confused either with an overall evaluation of conservation ARTs or with a general scheme for evaluating complex projects. This does not undermine its utility. The acceptability of the procedures—with respect to the mission of conservation, the welfare of the animals, the people involved, and the public opinion—is an important aspect to discriminate between those projects that are conducted responsibly and those that are not. As applications of conservation ART to endangered taxa will become more and more common, the need to explore their ethical implications becomes increasingly important.

The case study we analyzed is exemplary in this sense. Although the analysis is specifically built around the OPU procedures carried out on white rhinoceros in the context of the conservation efforts to save the NWR, the EM can be used as a template for analyzing ART procedures performed on other rhino taxa and other endangered species. It is rather plausible that the standard scenario of ART procedures administered to rhinoceros or other species for conservation efforts will be simpler than this case. However, this would not reduce the need to carefully address the ethical issues involved.

## Ethics Statement

The animal study was reviewed and approved by Internal Committee for Ethics and Animal Welfare of the Leibniz-IZW—Approval No: 2019-01-02.

## Author Contributions

PB, TH, and BM: conceptualization. PB: methodology, original draft writing and preparation, and visualization. CG and SH: data curation. PB, BM, TH, SH, FG, RH, CG, JS, IP, MS, GL, SC, SS, JZ, SN, SM, LK, IL, PO, and DN: editing and reviewing. BM: supervision and project administration. TH: fund acquisition. All authors contributed to the article and approved the submitted version.

## Funding

The publication of this article was supported by the Leibniz Association Open Access Fund and by the German Federal Ministry of Education and Research (BMBF) (BMBF BioRescue: 01LC1902A). The funders had no role in the study design, data collection and analysis, decision to publish, or preparation of the manuscript.

## Conflict of Interest

CG, GL, and SC were employed by company Avantea. The remaining authors declare that the research was conducted in the absence of any commercial or financial relationships that could be construed as a potential conflict of interest.

## Publisher's Note

All claims expressed in this article are solely those of the authors and do not necessarily represent those of their affiliated organizations, or those of the publisher, the editors and the reviewers. Any product that may be evaluated in this article, or claim that may be made by its manufacturer, is not guaranteed or endorsed by the publisher.
